# Inferring transcriptional bursting kinetics from single-cell snapshot data using a generalized telegraph model

**DOI:** 10.1098/rsos.221057

**Published:** 2023-04-05

**Authors:** Songhao Luo, Zhenquan Zhang, Zihao Wang, Xiyan Yang, Xiaoxuan Chen, Tianshou Zhou, Jiajun Zhang

**Affiliations:** ^1^ Guangdong Province Key Laboratory of Computational Science, Sun Yat-sen University, Guangzhou, Guangdong Province 510275, People's Republic of China; ^2^ School of Mathematics, Sun Yat-sen University, Guangzhou, Guangdong Province 510275, People's Republic of China; ^3^ School of Financial Mathematics and Statistics, Guangdong University of Finance, Guangzhou 510521, People's Republic of China

**Keywords:** inference, transcriptional bursting, single-cell snapshot, non-Markovian, gene expression

## Abstract

Gene expression has inherent stochasticity resulting from transcription's burst manners. Single-cell snapshot data can be exploited to rigorously infer transcriptional burst kinetics, using mathematical models as blueprints. The classical telegraph model (CTM) has been widely used to explain transcriptional bursting with Markovian assumptions. However, growing evidence suggests that the gene-state dwell times are generally non-exponential, as gene-state switching is a multi-step process in organisms. Therefore, interpretable non-Markovian mathematical models and efficient statistical inference methods are urgently required in investigating transcriptional burst kinetics. We develop an interpretable and tractable model, the generalized telegraph model (GTM), to characterize transcriptional bursting that allows arbitrary dwell-time distributions, rather than exponential distributions, to be incorporated into the ON and OFF switching process. Based on the GTM, we propose an inference method for transcriptional bursting kinetics using an approximate Bayesian computation framework. This method demonstrates an efficient and scalable estimation of burst frequency and burst size on synthetic data. Further, the application of inference to genome-wide data from mouse embryonic fibroblasts reveals that GTM would estimate lower burst frequency and higher burst size than those estimated by CTM. In conclusion, the GTM and the corresponding inference method are effective tools to infer dynamic transcriptional bursting from static single-cell snapshot data.

## 1. Introduction

Gene expression is a complex biochemical reaction process with inherent stochasticity, leading to cell-to-cell variability in messenger RNA (mRNA) abundance [1]. Many experimental studies have shown that, in both prokaryotic and eukaryotic cells, the expression of most genes exhibits a stochastic burst pattern over time, characterized by silent intervals interspersed between transcriptional events of genes [2,3]. Their burst kinetics, described by burst frequency and burst size [4,5], are closely related to the whole molecular processes of transcriptional regulation, but the mechanism is not clear. One crucial question is how to learn and infer interpretive biological mechanisms from extensive experimental data, thus bridging the disconnect between transcriptional bursts and their underlying molecular processes, which are crucial for understanding cell-fate decisions [6,7].

Addressing these questions requires visualizing transcription and measuring burst kinetics directly. A growing number of single-molecule experiments have dynamically highlighted transcriptional burst events. MS2 and PP7 imaging systems allow directly detecting the *in vivo* time-resolved RNA fluorescence of different genes within the same cell, revealing real-time dynamic transcriptional bursts in living cells [8–11]. Single-molecule fluorescence *in situ* hybridization (smFISH) [12,13] quantifies the steady-state distributions of RNA in thousands of fixed single cells, from which burst parameters such as burst frequency and burst size can be inferred. However, studies based on these experimental approaches are limited to a few genes, and the burst kinetics cannot be generalized to a genome-wide perspective. Recently, single-cell RNA sequencing (scRNA-seq) [14,15] has revolutionized our understanding of cell-fate decisions and made it possible to infer the dynamic behaviour of each gene from static expression distributions. To fulfil the promise of these scRNA-seq technologies, it will be crucial that mathematical models and computational methods are available to unambiguously reveal general principles of transcription on a genome-wide scale [4].

In principle, models of gene transcription should satisfy two basic requirements. First, the gene expression model should be interpretable and mechanistic. That is, the model can offer a way to understand the mechanisms behind transcriptional bursts—for example, addressing questions such as ‘how do the silent transcription intervals control transcriptional bursts?', or ‘how transcriptional bursting relates to gene regulation?’. Second, the model should be tractable. Tractability means the model can be analysed mathematically and used to infer transcriptional bursting kinetics for large datasets. The classical telegraph model (CTM) [16], the first rigorous mathematical treatment, connects transcription burst to stochastic gene expression. In this model, the gene switches randomly between active (ON) and inactive (OFF) states, with only the former permitting transcription initiation. The CTM has been applied in the genome-wide inference of burst kinetics from scRNA-seq [17–22]. For example, the inferences provided transcriptome-wide evidence that promoter elements affect burst size and enhancers control burst frequencies [17].

Despite the widespread use of the CTM, the model's basic assumption—gene switching between active and inactive states at constant rates—does not always hold in some specific biological systems [5,23,24]. Mathematically, the CTM is based on the Markovian assumption that all the biochemical reaction rates involved are constant, which implies that the dwell time in each state follows an exponential distribution [25]. However, most genes have complex control processes, such as chromatin opening, recruitment of transcription factors, pre-initiation complex formation, transcription initiation, as well as promoter pause and release [26]. Such processes could generate non-exponential time intervals between transcription windows in some genes or cell types. In particular, gene-state switching between active and inactive states is not a single-step manner but a multi-step process [24,27]. The multi-step process including sufficient rate-limiting steps can form a molecular memory between individual biochemical events [28], confirmed by increasing time-resolved biological experimental data [29,30]. Furthermore, this molecular memory can affect transcriptional burst kinetics [31,32].

Modelling, analysing and inferring the molecular memory in gene-state switching is challenging. One possible way is to introduce multiple intermediate states, i.e. the promoter architecture with multiple OFF and ON states [23,33,34]. Although the inclusion of additional gene states can improve the fit between a model and experimental data [35–37], the difficulty in determining a possibly large number of promoter states and parameters will be detrimental to the inference of the data [5,38–40]. Even though the topology structure of state-switching networks is simple, the number of transition rate parameters is the same order of the number of promoter states. Thus, it is difficult to handle in practical applications using a multi-state model in the case of numerous promoter states. Alternatively, one can adopt a non-Markovian modelling framework by introducing two general dwell-time distributions for OFF and ON states, respectively. Importantly, the general dwell-time distributions are not limited to the exponential distribution [28,41–45]. This non-Markovian model has two key advantages. First, the model is built in terms of experimentally measurable quantities and interpretable parameters, rather than unobserved or inconvenient measurement gene states. Second, the model can choose an appropriate distribution with fewer parameters to characterize the transition dynamics between only two gene states and therefore overcomes the difficulty in determining the number of promoter states. Despite the good properties of the non-Markovian model, it remains challenging to derive analytical solutions, develop a practical inference algorithm, and particularly infer bursting kinetics from scRNA-seq data. Our previous study described the gene expression process with the non-exponential dwell time of OFF state and exponential dwell time of ON state using a non-Markovian model [46]. However, there is experimental evidence that the non-exponential waiting time for the ON state is also important [47], and genome-wide inference has not been studied for gene expression models in which both OFF and ON dwell times are non-exponential.

In this study, we develop a statistical inference framework to infer transcriptional bursting kinetics from single-cell snapshot data based on a generalized telegraph model (GTM) we build that extends the traditional exponential dwell-time distributions for ON and OFF states to arbitrary distributions. We solve the model analytically and derive the arbitrarily high-order steady-state binomial moments for mRNAs. Furthermore, we develop a statistical inference method based on approximate Bayesian computation to estimate the burst kinetics of the GTM. As a result, we show that the CTM can be misleading for inferring burst kinetics from simulation data based on the GTM. After the validation of synthetic data, the results with our inference algorithm are accurate and scalable. Finally, we apply this dynamic model and inference method to scRNA-seq data from mouse embryonic fibroblasts and find that GTM which consider molecular memory would estimate lower burst frequency and higher burst size than those estimated by CTM on a genome-wide scale. In conclusion, our study provides a paradigm for inferring the transcriptional bursting kinetics from single-cell snapshot data.

## 2. Model

### 2.1. Model description

**Figure 1.**
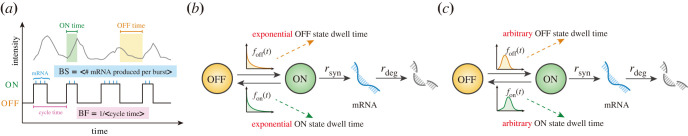
Schematic diagram burst kinetics and gene expression models. (*a*) The top panel shows a typical output of transcriptional burst in the mRNA production. The green shadow represents the time window of ON dwell time in transcription, while the orange shadow represents OFF dwell time. Correspondingly, the bottom panel represents the burst process in two states switching with each other. The short blue lines represent the transcription events during the ON state, and the purple line represents the cycle time (sum of ON and OFF time in one burst). Burst size (BS) is defined as the mean number (#) of mRNA produced per burst, and burst frequency (BF) is defined as the reciprocal of the mean cycle time. (*b*) The classical telegraph model (CTM), in which the promoter contains one OFF state and one ON state. The dwell time of the gene in these two states follows exponential distributions. The rates of mRNA synthesis and degradation are constants *r*_syn_ and *r*_deg_. (*c*) The generalized telegraph model (GTM), in which the promoter contains one OFF state and one ON state. The dwell time of the gene in these two states follows arbitrary distributions *f*_off_(*t*) and *f*_on_(*t*). The rates of mRNA synthesis and degradation are constants *r*_syn_ and *r*_deg_.

Transcription occurs predominantly in episodic bursts, characterized by burst frequency and burst size (figure 1*a*). The CTM is the prevailing model for describing the kinetic behaviour of transcriptional bursts (figure 1*b*). However, the promoter-state switching involves multiple biochemical reaction processes [48], resulting in the number of effective states of most promoters being greater than 2 (i.e. multiple ON states and OFF states) and diverse switching between states [5,23,33,39] (electronic supplementary material, figure S1). Mapping this complex promoter architecture to the ON–OFF non-Markovian model leads to the ON and OFF dwell times being no longer exponentially distributed, as reported in previous studies [24,27,37,49].

To make this idea precise, we consider a more general stochastic transcription model, called GTM, as illustrated in figure 1*c*. We assume that the dwell times in OFF and ON states, two random variables *τ*_off_ and *τ*_on_, follow arbitrary probability distributions, denoted by *f*_off_(*t*) and *f*_on_(*t*), rather than the limited exponential distributions. The mRNA synthesis and degradation process are assumed to be Markovian, i.e. exponential distributions of transcription waiting time *f*_syn_(*t*) and mRNA's lifetime *f*_deg_(*t*). Specifically, $f_{\mathrm{syn}}(t)=r_{\mathrm{syn}}e^{-r_{\mathrm{syn}}t}$, $f_{\deg}(t)=r_{\deg}e^{-r_{\deg}t}$, where *r*_syn_ and *r*_deg_ are the mean rate of mRNA synthesis and degradation, respectively. The reaction scheme is summarized by the reaction diagram:

2.1 $$\begin{array}{rl} & \mathrm{OFF}\overset{\;f_{\mathrm{off}}(t)}{⟶}\mathrm{ON},\mathrm{ON}\overset{\;f_{\mathrm{on}}(t)}{⟶}\mathrm{OFF},\\ & \mathrm{ON}\overset{r_{\mathrm{syn}}}{⟶}\mathrm{ON}+\mathrm{mRNA},\mathrm{mRNA}\overset{r_{\deg}}{⟶}\emptyset . \end{array}$$

### 2.2. Burst kinetic of the generalized telegraph model

The burst size and burst frequency (or the burst cycle time) are the two most critical parameters to characterize burst kinetics. First, we derive the probability distributions and their statistics for the burst size and cycle time with the GTM (figure 1*a*). Burst frequency describes the average number of bursts that occurred per unit time, i.e. the reciprocal of the mean cycle time. According to the definition of cycle time, which is the summation of the *τ*_off_ and *τ*_on_, the distribution of cycle time is the convolution of the distributions *f*_off_(*t*) and *f*_on_(*t*), i.e. $\left(\;f_{\mathrm{off}}\times f_{\mathrm{on}})(t\right)$. Consequently, we can obtain the expression of burst frequency, BF for GTM (see the electronic supplementary material, note S1.1 for a detailed derivation):

2.2 $$\mathrm{BF}=\frac{1}{\left\langle \tau_{\mathrm{off}}\right\rangle +\left\langle \tau_{\mathrm{on}}\right\rangle },$$

where $\left\langle \tau_{\mathrm{off}}\right\rangle =\int_{0}^{\infty }tf_{\mathrm{off}}(t)\;dt$ and $\left\langle \tau_{\mathrm{on}}\right\rangle =\int_{0}^{\infty }tf_{\mathrm{on}}(t)\;dt$ are the mean OFF and ON state dwell time, respectively.

Burst size describes the average number of mRNA molecules produced per burst. We first derive the distribution of transcription-event numbers per burst. For the exponential transcription process with rate *r*_syn_, the probability of the occurrence of *x* transcription events conditioned on a fixed duration time *t* of the ON state is a time-dependent Poisson distribution $P(X=x|\tau_{\mathrm{on}}=t)={\left(r_{\mathrm{syn}}t\right)}^{x}e^{-r_{\mathrm{syn}}t}/x!$. Then the probability of the transcription-event number per ON state period, denoted by *P*(*X*), can be computed by the total probability theorem $P(X=x)=\int_{0}^{\infty }P(X=x|\tau_{\mathrm{on}}=t)f_{\mathrm{on}}(t)\;dt$. Therefore, we obtain the burst size, BS for GTM by some algebraic calculations (see the electronic supplementary material, note S1.2 for a detailed derivation):

2.3 $$\mathrm{BS}=r_{\mathrm{syn}}\left\langle \tau_{\mathrm{on}}\right\rangle .$$

**Figure 2.**
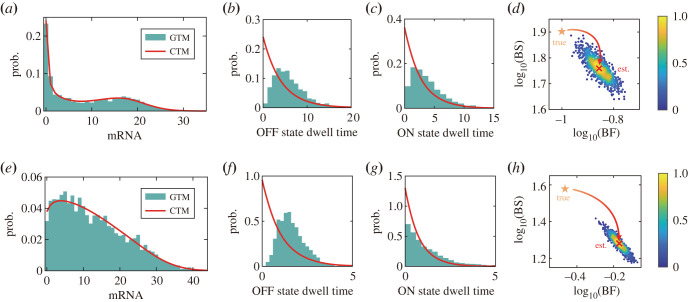
The CTM fits well the data generated by the GTM, but misleads the burst kinetics defined by GTM. (*a*) The histogram represents the data generated from the GTM with parameters *k*_off_ = 3, *r*_off_ = 0.5, *k*_on_ = 2, *r*_on_ = 0.5, *r*_syn_ = 20, *r*_deg_ = 1. The solid red line represents the mRNA distribution estimated from the CTM. (*b*) The histogram shows the simulated data of OFF-state dwell time from the GTM. The solid red line represents the distribution of OFF-state dwell time estimated from the CTM. (*c*) Results similar to (*b*) corresponds to the ON-state dwell time. (*d*) Scatter plots of transcriptional burst frequency (BF) and burst size (BS) were obtained from 1000 repeats of GTM simulations and CTM estimations. The red cross represents the most probable parameters obtained by using smooth kernel density for the burst parameters estimated by the CTM, while the orange star represents the true burst parameters calculated from the GTM. (*e–h*) The results of another example with the parameters *k*_off_ = 5, *r*_off_ = 3, *k*_on_ = 1, *r*_on_ = 0.8, *r*_syn_ = 30, *r*_deg_ = 1.

Next, we confirm the necessity of the GTM with the help of the obtained burst kinetics (equations (2.2) and (2.3)) of the GTM. From the principle of parsimony, we know that the burst size and frequency can also be directly computed with the CTM [17–19]. A natural and important question is whether the CTM quantitatively enough describes the burst kinetics (burst size and burst frequency). We answer it by performing inference on simulation data from the GTM. First, we repeatedly generate synthetic scRNA-seq data using the simulation algorithm of GTM (see the electronic supplementary material, table S1). In the GTM, molecular memories are characterized by Gamma distributions [31], i.e. $f_{\mathrm{off}}(t)=r_{\mathrm{off}}^{k_{\mathrm{off}}}t^{k_{\mathrm{off}}-1}e^{-r_{\mathrm{off}}t}/\Gamma (k_{\mathrm{off}})$ and $f_{\mathrm{on}}(t)=r_{\mathrm{on}}^{k_{\mathrm{on}}}t^{k_{\mathrm{on}}-1}e^{-r_{\mathrm{on}}t}/\Gamma (k_{\mathrm{on}})$ where Γ( · ) is Gamma function, *r*_off_ and *r*_on_ are the rate parameters of state switching and *k*_off_ and *k*_on_ are the number of possible reaction steps from OFF to ON and vice versa. The Gamma distribution with only two free parameters is widely used to characterize non-exponential dynamics in biophysical reality [29,50–52]. In addition, the Gamma distribution can be used to discern the existence of complex multi-step events in single-molecule data [53]. Other distributions that may be used are the Weibull distribution and so on. Then, we use the CTM to estimate the burst kinetic parameters of the synthetic data via the maximum-likelihood estimation approach. As a result, we find that although the CTM can well fit the gene expression distributions generated by the GTM (figure 2*a*,*e*), it cannot accurately describe the dwell-time distributions (figure 2*b*,*c*,*f*,*g*), further leading to the erroneous estimations of burst frequency and burst size defined by GTM (figure 2*d*,*h*). A recent work [54] has found that the different waiting time distributions between two mRNA production events can generate indistinguishable mRNA distributions. They pointed out that an analytical necessary condition should be satisfied to reduce the more detailed mechanistic model to the CTM. These results supported our study and suggest that when we use the non-time-resolved single-cell snapshots data to infer burst kinetics, different dwell-time distributions may yield identical gene expression distributions, which leads to misunderstandings of burst kinetics inferred from the CTM. Therefore, it is necessary to develop a scalable statistical inference approach to accurately estimate the burst kinetics from genome-wide scRNA-seq data based on the GTM.

### 2.3. Model analysis

To perform statistical inference on the burst kinetics by a steady gene expression distribution from static scRNA-seq data, we theoretically solve the statistical properties of the transcriptional process described in equation (2.1) using a supplementary variable method [55,56]. Denote by *M*(*t*) the number of mRNA molecules at time *t* and *G*(*t*) the gene state at time *t*. *E*_off_(*t*) (*E*_on_(*t*)) is defined as the elapsed time since the gene switches to the OFF (ON) state at time *t*, respectively. Then, {*M*(*t*), *G*(*t*), *E*_off_(*t*), E_on_(*t*);*t* ≥ 0} is a continuous-time Markov process. Let *p*_off_(*n*, *τ*, *t*) and *p*_on_(*n*, *τ*, *t*) be the probability density functions (PDF) that *n* mRNA molecules are produced and the elapsed time is *τ* in OFF and ON state at time *t*, respectively, and we have:

2.4 $$\begin{array}{rl} & p_{\mathrm{off}}(n,\tau ,t)\Delta \tau =\mathrm{Pr}\{N(t)=n,G(t)=\mathrm{OFF},\tau <E_{\mathrm{off}}(t)\leq \tau +\Delta \tau \},\\ & p_{\mathrm{on}}(n,\tau ,t)\Delta \tau =\mathrm{Pr}\{N(t)=n,G(t)=\mathrm{ON},\tau <E_{\mathrm{on}}(t)\leq \tau +\Delta \tau \}. \end{array}$$

According to the relationship between the states of the system at time *t* and *t* + Δ*t*, we obtain the following Chapman–Kolmogorov backward equations:

2.5 $$\begin{array}{c} \;p_{\mathrm{off}}(n,\;\tau +\Delta t,\;t+\Delta t)=p_{\mathrm{off}}(n,\;\tau ,\;t)(1-nr_{\deg}\Delta t)(1-H_{\mathrm{off}}(\tau )\Delta t)\\ +p_{\mathrm{off}}(n+1,\;\tau ,\;t)(n+1)r_{\deg}\Delta t(1-H_{\mathrm{off}}(\tau )\Delta t)+o(\Delta t),\\ p_{\mathrm{on}}(n,\;\tau +\Delta t,\;t+\Delta t)=p_{\mathrm{on}}(n,\;\tau ,\;t)(1-nr_{\deg}\Delta t)(1-r_{\mathrm{syn}}\Delta t)(1-H_{\mathrm{on}}(\tau )\Delta t)\\ +p_{\mathrm{on}}(n+1,\;\tau ,\;t)(n+1)r_{\deg}\Delta t(1-r_{\mathrm{syn}}\Delta t)(1-H_{\mathrm{on}}(\tau )\Delta t)\\ +p_{\mathrm{on}}(n-1,\;\tau ,\;t)(1-nr_{\deg}\Delta t)r_{\mathrm{syn}}\Delta t(1-H_{\mathrm{on}}(\tau )\Delta t)+o(\Delta t), \end{array}$$

where *H*_off_(*τ*) = *f*_off_(*τ*)/*S*_off_(*τ*) is a hazard rate function with the survival function $S_{\mathrm{off}}(\tau )=\int_{\tau }^{\infty }\;f_{\mathrm{off}}(t)\;dt$. The definitions of *H*_on_(*τ*) and *S*_on_(*τ*) are similar.

Next, we focus on steady-state distributions. If the stationary distributions of *p*_off_(*n*, *τ*, *t*) and *p*_on_(*n*, *τ*, *t*) exist (above simulation has verified this point), and are denoted by *p*_off_(*n*, *τ*) and *p*_on_(*n*, *τ*), respectively, equation (2.5) converts to the following stationary chemical master equation in the limit of small Δ*t* and large *t*:

2.6 $$\begin{array}{rl} & \frac{\partial }{\partial \tau }p_{\mathrm{off}}(n,\tau )=(n+1)r_{\deg}p_{\mathrm{off}}(n+1,\tau )-(nr_{\deg}+H_{\mathrm{off}}(\tau ))p_{\mathrm{off}}(n,\tau ),\\ & \frac{\partial }{\partial \tau }p_{\mathrm{on}}(n,\tau )=(n+1)r_{\deg}p_{\mathrm{on}}(n+1,\tau )+r_{\mathrm{syn}}p_{\mathrm{on}}(n-1,\tau )-(nr_{\deg}+r_{\mathrm{syn}}+H_{\mathrm{on}}(\tau ))p_{\mathrm{on}}(n,\tau ), \end{array}$$

with the integral boundary conditions $p_{\mathrm{off}}(n,0)=\int_{0}^{\infty }\;p_{\mathrm{on}}(n,\tau )H_{\mathrm{on}}(\tau )\;d\tau$, $p_{\mathrm{on}}(n,0)=\int_{0}^{\infty }\;p_{\mathrm{off}}(n,\tau )H_{\mathrm{off}}(\tau )\;d\tau$.

Based on equation (2.6) with its boundary conditions, we use the binomial moment method [57] to calculate the mRNA stationary distribution $P(n)=\int_{0}^{\infty }[\;p_{\mathrm{off}}(n,\tau )+p_{\mathrm{on}}(n,\tau )]\;d\tau$ and its statistical characteristics. Binomial moments of the mRNA stationary distribution are defined as $b_{n}=\sum_{m\geq n}^{\infty }\left(\begin{array}{c} m\\ n \end{array}\right)P(m)$, where the symbol $\left(\begin{array}{c} m\\ n \end{array}\right)$ represents the combinatorial number. Note that binomial moments converge to zero as their orders go to infinity, and can be used to reconstruct *P*(*n*) by $P(n)=\sum_{m=n}^{\infty }{\left(-1\right)}^{m-n}\left(\begin{array}{c} m\\ n \end{array}\right)b_{m}$. After some algebra of equation (2.6), we can obtain the *n*th binomial moment of mRNA in a recursive form (see the electronic supplementary material, note S2 for a detailed derivation):

2.7 $$b_{n}=\frac{1}{n!}{\left(\frac{r_{\mathrm{syn}}}{r_{\deg}}\right)}^{n}\sum_{i=0}^{n-1}\left(\begin{array}{c} n-1\\ i \end{array}\right)C_{i}\sum_{\;j=0}^{n-1-i}\left(\begin{array}{c} n-1-i\\ j \end{array}\right){\left(-1\right)}^{n-1-i-j}{\tilde{S}}_{\mathrm{on}}((n-1-j)r_{\deg}),$$

where

2.8 $$C_{n}=\frac{{\tilde{\;f}}_{\mathrm{off}}(nr_{\deg})}{1-{\tilde{\;f}}_{\mathrm{off}}(nr_{\deg}){\tilde{\;f}}_{\mathrm{on}}(nr_{\deg})}\sum_{i=0}^{n-1}\left(\begin{array}{c} n\\ i \end{array}\right)C_{i}\sum_{\;j=0}^{n-i}\left(\begin{array}{c} n-i\\ j \end{array}\right){\left(-1\right)}^{n-i-j}{\tilde{\;f}}_{\mathrm{on}}((n-j)r_{\deg}),$$

for *n* = 1, 2, …. Here, *C*_0_ = (〈*τ*_off_〉 + 〈*τ*_on_〉)^−1^ is equal to the burst frequency. $\tilde{\;f}(s)$ represents the Laplace transform of function *f*(*t*). Especially, ${\tilde{S}}_{\mathrm{on}}(s)=\left(1-{\tilde{\;f}}_{\mathrm{on}}(s)\right)/s$ and ${\tilde{S}}_{\mathrm{on}}(0)=\left\langle \tau_{\mathrm{on}}\right\rangle$. According to the relationship between binomial moments and central moments and equations (2.7) and (2.8), we obtain the mean and noise of mRNA expression:

2.9 $$\mathrm{mean}=\frac{r_{\mathrm{syn}}\left\langle \tau_{\mathrm{on}}\right\rangle }{r_{\deg}\left(\left\langle \tau_{\mathrm{off}}\right\rangle +\left\langle \tau_{\mathrm{on}}\right\rangle \right)}$$

and

2.10 $${\mathrm{CV}}^{2}=\frac{1}{\mathrm{mean}}+\frac{\left\langle \tau_{\mathrm{off}}\right\rangle }{\left\langle \tau_{\mathrm{on}}\right\rangle }-\frac{\left\langle \tau_{\mathrm{off}}\right\rangle +\left\langle \tau_{\mathrm{on}}\right\rangle }{r_{\deg}{\left\langle \tau_{\mathrm{on}}\right\rangle }^{2}}\frac{\left(1-{\tilde{\;f}}_{\mathrm{off}}(r_{\deg}))(1-{\tilde{\;f}}_{\mathrm{on}}(r_{\deg})\right)}{1-{\tilde{\;f}}_{\mathrm{off}}(r_{\deg}){\tilde{\;f}}_{\mathrm{on}}(r_{\deg})}.$$

Note that if let *τ*_off_*r*_deg_ and *τ*_on_*r*_deg_ be the rescaled random variables for OFF-state and ON-state dwell times, and *r*_syn_/*r*_deg_ be the rescaled mean synthesis rate, the mean expression not only equals the product of the mean synthesis rate and the stationary probability of ON state, but also the product of burst size and burst frequency.

Finally, we consider two specific cases of the GTM. First, if the ON-state dwell time is exponential, i.e. $f_{\mathrm{on}}(t)=e^{-t/\left\langle \tau_{\mathrm{on}}\right\rangle }/\left\langle \tau_{\mathrm{on}}\right\rangle$, the GTM and corresponding results reduce to the previous conclusions [46] (see the electronic supplementary material, note S3.1). Further, if the OFF-state dwell time is also exponential, i.e. $f_{\mathrm{off}}(t)=r_{\mathrm{off}}e^{-r_{\mathrm{off}}t}$, the GTM reduces to the CTM. In this model, the mean activation and inactivation rates are *r*_off_ = 〈*τ*_off_〉^−1^ and *r*_on_ = 〈*τ*_on_〉^−1^, respectively. Notably, the expression for the *n*th binomial moment of mRNA can be simplified as follows (see the electronic supplementary material, note S3.2):

2.11 $$b_{n}=\frac{{\left(r_{\mathrm{syn}}r_{\deg}^{-1}\right)}^{n}}{n!}\frac{\Gamma (r_{\mathrm{on}}r_{\deg}^{-1}+n)}{\Gamma (r_{\mathrm{on}}r_{\deg}^{-1})}\frac{\Gamma (r_{\mathrm{on}}r_{\deg}^{-1}+r_{\mathrm{off}}r_{\deg}^{-1})}{\Gamma (r_{\mathrm{on}}r_{\deg}^{-1}+r_{\mathrm{off}}r_{\deg}^{-1}+n)}.$$

Using the reconstruct formula, we obtain the mRNA stationary distribution for CTM (see the electronic supplementary material, note S3.2), consistent with previous results [3,16].

## 3. Binomial moment-based inference

In this section, we will develop a statistical inference method based on binomial moments for GTM. First, we assume that the number of cells in the experiment is large enough to allow us to acquire a steady expression distribution of a specific gene. This assumption is reasonable in scRNA-seq data and has been widely applied to genome-wide studies [17,20]. Next, with the steady distribution of each gene from scRNA-seq data as a bridge, we develop a computation inference framework that uses an approximate Bayesian computation (ABC) approach [58] to infer reliable parameter sets for our GTM (see the electronic supplementary material, table S2).

### 3.1. Inference framework

We develop a statistical framework that combines the ABC approach to estimate the Bayesian posterior probability *π*(**θ**|**y**_obs_) of the model parameter-vector (**θ**) given the observed scRNA-seq data (**y**_obs_). The prior information related to **θ** is denoted as the prior distribution *π*(**θ**), which will be iteratively updated through computing the likelihood function *p*(**y**_obs_|**θ**) of the GTM. Using Bayes' theorem, the resulting posterior distribution is given by:

3.1 $$\pi (\theta |y_{\mathrm{obs}})=\frac{\;p(y_{\mathrm{obs}}|\theta )\pi (\theta )}{\int_{\Theta }\;p(y_{\mathrm{obs}}|\theta )\pi (\theta )d\theta }.$$

Ideally, we can perform the inference methods relying on iterative likelihood function maximization. However, the approximation of the mRNA distribution of the GTM is computationally prohibitive, making it impossible to evaluate the likelihood function directly.

**Figure 3.**
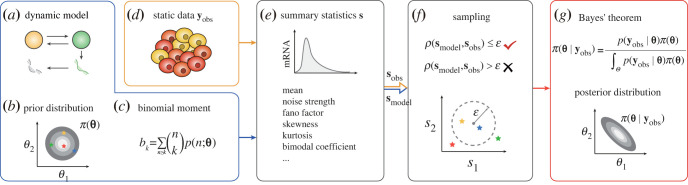
Inference procedure. Given a dynamic model (*a*), the parameter **θ** is sampled from the prior distribution *π*(**θ**) (*b*), and then the theoretical binomial moment (*c*) is used to compute the summary statistic **s**_model_ (*e*). The static single-cell snapshot data **y**_obs_ (*d*) can be used to calculate the summary statistic **s**_obs_ (*e*). The sampled **θ** is accepted by comparing whether *ρ*(**s**_obs_, **s**_model_) is less than the threshold *ε* (*f*). As the output of the inference procedure, the posterior distribution *π*(**θ**|**y**_obs_) of the parameters is obtained by Bayes' theorem (*g*).

Alternatively, we resort to a ‘likelihood-free' Bayesian approach by computing only low-order moments instead of the whole probability distribution according to the obtained binomial moments (equation (2.7)). Specifically, we use the sequential Monte Carlo ABC [59] (see the electronic supplementary material, table S2), a variant of ABC, to implement the statistical inference of our model. ABC allows us to accept the parameters that make the simulated data and the observed data sufficiently close in distribution and to estimate the posterior distribution *π*(**θ**|**y**_obs_) of the parameters through numerous simulations. First, given a dynamic model (figure 3*a*), we sample a candidate parameter-vector **θ** from the prior distribution *π*(**θ**) (figure 3*b*), and simulate a dataset **y**_model_ from the GTM. Then, we check whether the distribution of simulated data approximates the observed data **y**_obs_ (figure 3*d*) by predefining three extra parameters: (i) summary statistics **s**(**y**) for sufficiently representing data; (ii) discrepancy metrics *ρ*( · , · ) for measuring the distance between summary statistics of observed data **s**_obs_ and simulated data **s**_model_ from the GTM (figure 3*e*); and (iii) threshold *ε* for controlling acceptable errors (figure 3*f*). Note that the low threshold *ε* of ABC promises a good approximation of *π*(**θ**|**y**_obs_), but also imposes a huge computational cost and low-rate acceptances. To avoid the difficulties of ABC in terms of computational power and convergence, we use the sequential Monte Carlo ABC.

Sequential Monte Carlo ABC adds a sequence of threshold values {*ε*_0_, *ε*_1_, …, *ε_T_*} satisfied the condition *ε*_0_ > · · · > *ε_T_* ≥ 0, and thus constructs a sequence of the intermediate posterior distributions $\left\{\;f_{t}(\theta |\rho (s_{\mathrm{obs}},s_{\mathrm{model}})\leq \varepsilon_{t})\},t\in \{0,1,\ldots ,T\right\}$, where *f*_0_(**θ**) = *π*(**θ**). When the (*t* − 1)th iteration is over, we can obtain the *f_t_*_−1_(**θ**|*ρ*(**s**_obs_, **s**_model_) ≤ *ε_t_*_−1_). In the *t*th iteration, we first sample the parameters **θ***_t_*_−1_ randomly from *f_t_*_−1_(**θ**|*ρ*(**s**_obs_, **s**_model_) ≤ *ε_t_*_−1_) and sample a θ* from the proposal distribution *K_t_*(**θ**|**θ***_t_*_−1_). Then we accept the θ* if $\rho (s_{\mathrm{obs}},s_{\mathrm{model}}(\theta^{*}))\leq \varepsilon_{t}$. After repeating the above procedures in this iteration, we get the posterior distribution *f_t_*(**θ**|*ρ*(**s**_obs_, **s**_model_) ≤ *ε_t_*). In theory, $f_{T}(\theta |\rho (s_{\mathrm{obs}},s_{\mathrm{model}})\leq \varepsilon_{T})=\pi (\theta |y_{\mathrm{obs}})$ when *ε_T_* → 0. Finally, we can obtain the posterior distribution *π*(**θ**|**y**_obs_) (figure 3*g*).

### 3.2. Inference procedure

#### 3.2.1. Summary statistics

The inference procedure requires a summary statistic s(y) to reduce the high-dimensional data to low-dimension features to compare whether the distribution of simulated data **y**_model_ from the GTM is close to the observed data **y**_obs_. Here, we choose six commonly used moment statistics for inference [60,61] that are important to characterize the shape of gene expression distribution as the summary statistics: (i) the mean value *μ*_1_ is the most commonly used indicator in statistics, and it represents the average level of mRNA expression in scRNA-seq data; (ii) the noise strength is a measurement of the dispersion of the probability distribution, defined as $\mu_{2}/\mu_{1}^{2}$ where *μ*_2_ is the variance; (iii) the Fano factor is another statistic that measures the dispersion of a probability distribution relative to a Poisson distribution, defined as *μ*_2_/*μ*_1_; (iv) the skewness is a description of the symmetry of the distribution, and it is defined as $\mu_{3}/\mu_{2}^{3/2}$, where *μ*_3_ is the third central moment; (v) the kurtosis describes whether the peak of the distribution is abrupt or flat, which is defined as $\mu_{4}/\mu_{2}^{2}$, where *μ*_4_ is the fourth central moment; and (vi) the bimodality coefficient can describe the bimodal distribution [62], which is usually a critical feature in a dynamical system. The values of the bimodality coefficient range from 0 to 1, and values greater than 5/9 may indicate a bimodal or multimodal distribution.

Crucially, we used the binomial moment approach which theoretically provides an efficient method to directly compute the theoretical summary statistics for a given parameter **θ** of the GTM. Precisely, we can calculate the central moments with the binomial moment:

3.2 $$\mu_{k}(t)={\left(-b_{1}(t)\right)}^{k}+\sum_{i=0}^{k-1}\sum_{\;j=0}^{k-i}R(k,i,j)(\;j!){\left(b_{1}(t)\right)}^{i}b_{j}(t),$$

in which $R(k,i,j)={\left(-1\right)}^{i}\left(\begin{array}{c} k\\ i \end{array}\right)S(k-i,j)$ with $S(n,k)=\sum_{i=0}^{k}{\left(-1\right)}^{k-i}\left(\begin{array}{c} k\\ i \end{array}\right)i^{n}$ being the Stirling number of the second kind. Therefore, the above summary statistics can be expressed by binomial moments as follows:

3.3 $$\begin{array}{rl} & \mathrm{mean}=b_{1},\\ & \mathrm{noise}\;\mathrm{strength}=\frac{2b_{2}+b_{1}-b_{1}^{2}}{b_{1}^{2}},\\ & \mathrm{Fano}\;\mathrm{factor}=\frac{2b_{2}+b_{1}-b_{1}^{2}}{b_{1}},\\ & \mathrm{skewness}=\frac{6b_{3}+6b_{2}+b_{1}-3b_{1}(2b_{2}+b_{1})+2b_{1}^{3}}{{\left(2b_{2}+b_{1}-b_{1}^{2}\right)}^{3/2}},\\ & \mathrm{kurtosis}=\frac{24b_{4}+36b_{3}+14b_{2}+b_{1}-4b_{1}(6b_{3}+6b_{2}+b_{1})+6b_{1}^{2}(2b_{2}+b_{1})-3b_{1}^{4}}{{\left(2b_{2}+b_{1}-b_{1}^{2}\right)}^{2}}-3,\\ & \mathrm{bimodality}\;\mathrm{coefficient}=\frac{{\mathrm{skewness}}^{2}+1}{\mathrm{kurtosis}}. \end{array}$$

It should be noted that we can extend the summary statistics to higher-order moments because our binomial moments can compute arbitrary high-order moment statistics. However, we emphasize that the practice of calculating summary statistics directly from theory rather than through simulations works well but is quite unusual in the context of ABC. As a result, the algorithm is not a true Bayesian approach, because it makes the acceptance and rejection for a set of parameters become a deterministic process, and the width of the posterior distribution obtained by the algorithm will be narrower than the true posterior distribution. We suggested that the mode of the posterior distribution obtained by the algorithm is credible, while the error of the width is dependent on the sample size (the larger the sample size, the smaller the width error) [58].

To assess the sensitivity of the summary statistics, we investigate the influence of the model parameters (*k*_on_, *k*_off_, *r*_on_ and *r*_off_) on the six summary statistics through the simulation algorithm of GTM (electronic supplementary material, table S1) and the theoretical results (equation (3.3)). Note that the parameter of ON and OFF dwell times show opposite tendencies of influence on the summary statistics (electronic supplementary material, figures S2 and S3). The results also show that the six summary statistics are sensitive to the parameters of dwell times, implying the rationality of statistics selection.

#### 3.2.2. Discrepancy metrics

To eliminate the influence of absolute size between different summary statistics, we take the natural logarithm of the data instead of computing the Euclidean distance [36,63]:

3.4 $$\rho (s_{\mathrm{obs}},s_{\mathrm{model}})=\sqrt{\sum_{i=1}^{n}{\left(\log s_{\mathrm{obs}}^{\left(i\right)}-\log s_{\mathrm{model}}^{\left(i\right)}\right)}^{2}},$$

where **s**^(*i*)^ is the *i*th component of the summary statistics vector. Note that the logarithm transformations of data do not change the data properties and correlation, but the scale is compressed. In addition, these transformations can make the data more stable and weaken the effects of collinearity and heteroscedasticity on the model.

#### 3.2.3. Acceptance threshold

Generally, the acceptance thresholds {*ε*_0_, *ε*_1_, …, *ε_T_*} are usually determined by experience. However, the algorithm causes a waste of computational resources in this iteration if the default threshold is greater than the maximum distance obtained by sampling in the last iteration. Therefore, we adopt a strategy of adaptive acceptance thresholds to prevent this situation. For the first iteration, we use a large threshold *ε*_0_ to accept 10 000 coarse parameter samples quickly, and then select the first 1000 parameter samples with the smallest discrepancy between **s**_obs_ and **s**_model_ as input for the next iteration. For the *t*th iteration, the acceptance threshold *ε_t_* is set to the median of the discrepancies of the results obtained from the (*t* − 1)th iteration. In detail, we set the hyperparameters: initial thresholds *ε*_0_ = 1 and round number *T* = 5. With this setting, it takes about 75 s to run an example of synthetic data using an Intel(R) Core(TM) i7–6700 CPU @ 3.40 GHz.

#### 3.2.4. Prior distribution

As required in Bayesian inference, we should set prior distribution *π*(**θ**), allowing for the initial parameter sampling **θ** in the inference procedure. In the GTM, we assume that the mRNA decay rate *r*_deg_ = 1 and the dwell times in the ON state and OFF state are Gamma distributed, i.e. $f_{\mathrm{off}}(t)=r_{\mathrm{off}}^{k_{\mathrm{off}}}t^{k_{\mathrm{off}}-1}e^{-r_{\mathrm{off}}t}/\Gamma (k_{\mathrm{off}})$ and $f_{\mathrm{on}}(t)=r_{\mathrm{on}}^{k_{\mathrm{on}}}t^{k_{\mathrm{on}}-1}e^{-r_{\mathrm{on}}t}/\Gamma (k_{\mathrm{on}})$, respectively. The parameter-vector is **θ =** [*k*_off_, *r*_off_, *k*_on_, *r*_on_, *r*_syn_]. We set the prior distributions of *k*_off_ and *k*_on_ follow the uniform distribution *U*[0, 5] and the prior distributions of *r*_off_ and *r*_on_ follow the log-uniform distribution from interval [ − 1, 1] based on 10. In addition, the prior distribution of transcriptional rates *r*_syn_ is *U*[0, 50].

#### 3.2.5. Proposal distribution

In the inference procedure, the parameter θ* sampled in *t*th iteration is based on small perturbations around the parameter **θ***_t_*_−1_ sampled from the distribution *f_t_*_−1_(**θ**|*ρ*(**s**_obs_, **s**_model_) ≤ *ε_t_*_−1_). Therefore, we use lognormal distribution *LN*(**θ**;**θ***_t_*_−1_, *σ*) as the proposed distribution for sampling all parameters in the *t*th iteration. In detail, we set the hyperparameters: the varience of lognormal distribution *σ* = 0.2 for all parameter.

## 4. Results

### 4.1. Synthetic data

**Figure 4.**
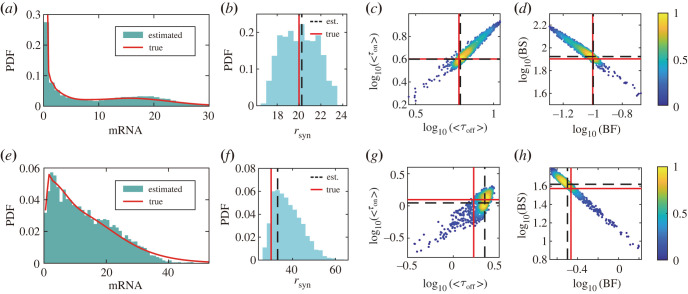
Validation of inferring burst kinetics on synthetic scRNA-seq data. (*a*) Comparison between synthetic scRNA-seq data for inference with parameters *k*_off_ = 3, *r*_off_ = 0.5, *k*_on_ = 2, *r*_on_ = 0.5, *r*_syn_ = 20, $r_{\deg}=1$ (red solid line, the PDF fitted by kernel smoothing function) and simulation data of inferred parameters (green histogram). (*b*) Marginal posterior distributions (blue histogram) of transcriptional rate *r*_syn_ estimated by the inference method, where the red solid line corresponds to the true parameter and the black dashed line to the mode of estimated parameter. (*c*) The scatter plot shows the two-dimensional posterior distribution of the dwell time *τ*_off_ and *τ*_on_. The colour bar is a normalized probability density. The meanings of lines are the same as those in (*b*). (*d*) Posterior distribution of the burst frequency (BF) and burst size (BS). (*e–h*) The results of another example with the parameters *k*_off_ = 5, *r*_off_ = 3, *k*_on_ = 1, *r*_on_ = 0.8, *r*_syn_ = 30, $r_{\deg}=1$.

We first use synthetic scRNA-seq data from the GTM to verify whether the inference method can accurately estimate the burst kinetics of the gene expression. We apply the simulation algorithm for the GTM with given parameters (see the electronic supplementary material, table S1) to generate mRNA distribution. Figure 4*a* shows that the dwell times for OFF and ON states for the GTM are non-exponential distributions and have a bimodal distribution at a steady state, which has important significance in biological systems [64] and is a common observation in scRNA-seq data [65]. Having obtained 1000 samples by the inference procedure, we find that the posterior distribution of transcriptional rate *r*_syn_ can accurately estimate the true parameter value (figure 4*b*). Importantly, we show that the dwell time's first-order moment information (mean) can be estimated accurately (figure 4*c*). In addition, the burst frequency and burst size can also be estimated accurately (figure 4*d*) (although the individual parameters are unidentifiable in large part of parameter space). In another example, the distribution of GTM is unimodal, and the burst kinetics can also be accurately inferred (figure 4*f–h*).

### 4.2. Experimental data

Next, we apply the inference procedure to mouse embryonic fibroblasts for each allele (C57 × CAST) [17] to estimate transcriptional bursting parameters on a genome-wide scale. This scRNA-seq data was widely used to investigate genome-wide transcriptional burst kinetics [20,66,67], which was sequenced based on smart-seq2 technology (unique molecular identifier counts were used) and contained 10 727 genes and 224 individual cells on each allele. For quality control, we filter out genes expressed in less than 50 cells and cells expressed in less than 2000 genes. In addition, we remove genes with mean expression levels below 2 to ensure high expression of the inferred genes. Finally, we combine the two allele expressions together to eventually form a single-cell matrix consisting of 2162 genes and 413 cells.

**Figure 5.**
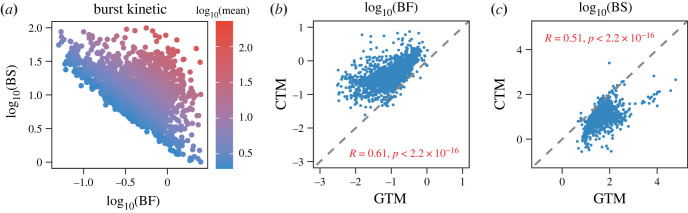
Genome-wide characteristics of transcriptional burst kinetics inferred from the scRNA-seq data of mouse embryonic fibroblasts. (*a*) Scatter plot of burst frequency (BF) and burst size (BS) inferred by the inference method, where the colour bar represents the mean expression levels of each gene. (*b*,*c*) Scatter plots show the burst frequency (*b*) and burst size (*c*) estimated by GTM and CTM, which are correlated in the sense of Pearson correlation test (*p*-value < 2.2 × 10^−16^). The slope of the grey dashed lines equals 1.

Interestingly, we observe that the genes with the same average expression level have a different combination of burst frequency and burst size, consistent with previous reports [17,24]. This result implies that gene expression may be regulated by diverse burst kinetic mechanisms (figure 5*a*). In addition, we perform genome-wide burst kinetics inference for the same data based on the CTM, using a maximum likelihood estimation method. We discover that the estimated burst frequency and burst size keep a high positive correlation between GTM and CTM (*p*-value < 2.2 × 10^−16^; figure 5*b*,*c*). Notably, we observe that the transcriptional burst kinetics inferred with the GTM would have lower burst frequency (figure 5*b*) and higher burst size (figure 5*c*) than those estimated by CTM on the genome-wide scale, consistent with the results for single genes in figure 2*d*,*h*. Moreover, the inference method with an alternative definition of the burst frequency (1/〈*τ*_off_〉) also presents the same results (electronic supplementary material, figure S4). These results suggest that the GTM, as an extension of the CTM, provide a more flexible way to predict burst kinetics, and corresponding inference methods has the capability to perform genome-wide studies on scRNA-seq data.

## 5. Discussion

With the emergence of next-generation sequencing technologies, inferring gene expression burst kinetics on a genome-wide scale from static single-cell snapshot data is challenging in computational systems biology [4]. In previous studies [17,18], gene expression models used for inference and analysis, e.g. CTM, have long relied on Markov assumptions. However, increasing experimental data show that the dwell time of states is not simply exponentially distributed [24,27,37,49], leading to the failure of the Markov approximation. There is thereby an urgent need but it is still a significant challenge to develop effective methods for modelling, analysing and inferring non-Markov gene expression models.

In this paper, we have developed an inference method for inferring burst kinetics from scRNA-seq data based on GTM, which allows consideration of ON–OFF transitions with arbitrary dwell-time distributions. We demonstrated that the CTM could not estimate the non-Markovian burst kinetics, although it can well fit the gene expression distributions sometimes. We theoretically derived the analytical solution for arbitrary order binomial moments of GTM, which in turn enables us to calculate the statistics of mRNA. We developed the inference procedure to infer transcriptional burst kinetics using the summary statistic calculated by binomial moments. The results of the synthetic dataset show that our inference method can precisely estimate the burst frequency and burst size of the gene expression system as well as the average dwell time in ON and OFF states based on GTM. Furthermore, we performed a genome-wide burst kinetics inference on the mouse embryonic fibroblasts scRNA-seq data with the inference method. We found that the transcriptional burst kinetics inferred with the GTM would have lower burst frequency and higher burst size than CTM.

The GTM and the corresponding inference method are applicable to study burst kinetics on a genome-wide scale. First, the GTM is interpretable. The GTM, as an extension of the CTM, is a mechanistic model that considers the dwell times to be arbitrary distributions; and the model parameters, such as dwell times of OFF and ON states, transcription rates and degradation rates, are measurable from experiments [7,24]. Second, the GTM is solvable. The arbitrary order binomial moments of mRNA's distribution are theoretically derived. In particular, the model extends previous results. For example, the CTM is a special case of the GTM if *f*_off_(*t*) and *f*_on_(*t*) are set to exponential distributions. Third, the inference method for GTM is scalable. The approximation of the probability distributions of the stochastic model is often computationally prohibitive. With the theoretical results derived, we used a ‘likelihood-free' approach by computing only low-order moments instead of the whole probability distribution. The efficiency of the inference method facilitates the extension of individual genes to genome-wide study. Finally, our inference method can learn gene expression information from static snapshot data without time-resolved data.

Our work opens up several avenues for further research. From a modelling perspective, the GTM simplifies biological processes in several aspects. The GTM only considers the generalized dwell-time distribution for OFF and ON states but does not consider the non-exponential waiting time for transcriptional and degradation processes. Many experimental data suggest that transcription (such as mRNA elongation, pause and release [68–70]) and degradation (such as mRNA senescence [31,71,72]) are also multi-step processes in cells. In addition, other biological processes that can affect burst kinetics should be considered in future work. For example, a recent study reveal that the inference of splicing dynamics can further investigate the transcriptional burst dynamics [73]. Also, another study has investigated that the post-transcriptional noise and different cell cycle phases would effect the transcription inference [74]. From a statistical inferring perspective, using GTM to interpret the experimental biological data has yet to be fully developed. First, the binomial moments obtained from our analysis are in the sense of a steady distribution (*t* → ∞) and not as a function of time *t*. Solving for the model's temporal solutions facilitates applying the GTM to time-resolved data. Second, the inference based on the steady-state distribution still suffers from the unidentifiability of the parameters, which may depend on the properties that different dwell-time distributions lead to the same static mRNA distribution. Third, a fraction of the variability in scRNA-seq data comes from technical noise [75], and it is a challenge to couple the technical noise into the GTM for inference, owing to the computational complexity of the model. Inspired by recent studies [20,76], we can address this issue in our future work by introducing sampling distributions.

Finally, we note that our approach is not limited to scRNA-seq data, but could also be useful for other kinds of single-cell data in which the probability distributions of mRNA can be estimated, such as smFISH data. We expect the GTM and corresponding inference method to facilitate an understanding of gene expression mechanisms from the enormous amount of biological data.

## Data Availability

Relevant code of algorithm and analysis for this research work are stored in GitHub: https://github.com/cellfate/BurstGTM and have been archived within the Zenodo repository: https://doi.org/10.5281/zenodo.7512499. Experimental data of mouse primary fibroblasts were downloaded from [17]. Additonal data is available in the electronic supplementary material [77].

## References

[1] Elowitz MB, Levine AJ, Siggia ED, Swain PS. 2002 Stochastic gene expression in a single cell. Science **297**, 1183-1186. (10.1126/science.1070919) 12183631

[2] Yu J, Xiao J, Ren X, Lao K, Xie XS. 2006 Probing gene expression in live cells, one protein molecule at a time. Science **311**, 1600-1603. (10.1126/science.1119623) 16543458

[3] Raj A, Peskin CS, Tranchina D, Vargas DY, Tyagi S. 2006 Stochastic mRNA synthesis in mammalian cells. PLoS Biol. **4**, e309. (10.1371/journal.pbio.0040309) 17048983 PMC1563489

[4] Rodriguez J, Larson DR. 2020 Transcription in living cells: molecular mechanisms of bursting. Annu. Rev. Biochem. **89**, 189-212. (10.1146/annurev-biochem-011520-105250) 32208766

[5] Tunnacliffe E, Chubb JR. 2020 What is a transcriptional burst? Trends Genet. **36**, 288-297. (10.1016/j.tig.2020.01.003) 32035656

[6] Eldar A, Elowitz MB. 2010 Functional roles for noise in genetic circuits. Nature **467**, 167-173. (10.1038/nature09326) 20829787 PMC4100692

[7] Lammers NC, Kim YJ, Zhao J, Garcia HG. 2020 A matter of time: using dynamics and theory to uncover mechanisms of transcriptional bursting. Curr. Opin. Cell Biol. **67**, 147-157. (10.1016/j.ceb.2020.08.001) 33242838 PMC8498946

[8] Chen SY et al. 2020 Optogenetic control reveals differential promoter interpretation of transcription factor nuclear translocation dynamics. Cell Syst. **11**, 336-353.e24. (10.1016/j.cels.2020.08.009) 32898473 PMC7648432

[9] Chao JA, Patskovsky Y, Almo SC, Singer RH. 2008 Structural basis for the coevolution of a viral RNA-protein complex. Nat. Struct. Mol. Biol. **15**, 103-105. (10.1038/nsmb1327) 18066080 PMC3152963

[10] Bertrand E, Chartrand P, Schaefer M, Shenoy SM, Singer RH, Long RM. 1998 Localization of ASH1 mRNA particles in living yeast. Mol. Cell **2**, 437-445. (10.1016/S1097-2765(00)80143-4) 9809065

[11] Larson DR, Zenklusen D, Wu B, Chao JA, Singer RH. 2011 Real-time observation of transcription initiation and elongation on an endogenous yeast gene. Science **332**, 475-478. (10.1126/science.1202142) 21512033 PMC3152976

[12] Femino AM, Fay FS, Fogarty K, Singer RH. 1998 Visualization of single RNA transcripts *in situ*. Science **280**, 585-590. (10.1126/science.280.5363.585) 9554849

[13] Raj A, Van Den Bogaard P, Rifkin SA, Van Oudenaarden A, Tyagi S. 2008 Imaging individual mRNA molecules using multiple singly labeled probes. Nat. Methods **5**, 877-879. (10.1038/nmeth.1253) 18806792 PMC3126653

[14] Zheng GX et al. 2017 Massively parallel digital transcriptional profiling of single cells. Nat. Commun. **8**, 1-12. (10.1038/ncomms14049) 28091601 PMC5241818

[15] Picelli S, Björklund ÅK, Faridani OR, Sagasser S, Winberg G, Sandberg R. 2013 Smart-seq2 for sensitive full-length transcriptome profiling in single cells. Nat. Methods **10**, 1096-1098. (10.1038/nmeth.2639) 24056875

[16] Peccoud J, Ycart B. 1995 Markovian modeling of gene-product synthesis. Theor. Popul. Biol. **48**, 222-234. (10.1006/tpbi.1995.1027)

[17] Larsson AJ et al. 2019 Genomic encoding of transcriptional burst kinetics. Nature **565**, 251-254. (10.1038/s41586-018-0836-1) 30602787 PMC7610481

[18] Kim JK, Marioni JC. 2013 Inferring the kinetics of stochastic gene expression from single-cell RNA-sequencing data. Genome Biol. **14**, R7. (10.1186/gb-2013-14-1-r7) 23360624 PMC3663116

[19] Ochiai H et al. 2020 Genome-wide kinetic properties of transcriptional bursting in mouse embryonic stem cells. Sci. Adv. **6**, eaaz6699. (10.1126/sciadv.aaz6699) 32596448 PMC7299619

[20] Luo S, Wang Z, Zhang Z, Zhou T, Zhang J. 2022 Genome-wide inference reveals that feedback regulations constrain promoter-dependent transcriptional burst kinetics. Nucleic Acids Res. **11**, 68-83. (10.1093/nar/gkac1204) PMC9874261 36583343

[21] Vu TN, Wills QF, Kalari KR, Niu N, Wang L, Rantalainen M, Pawitan Y. 2016 Beta-Poisson model for single-cell RNA-seq data analyses. Bioinformatics **32**, 2128-2135. (10.1093/bioinformatics/btw202) 27153638 PMC13048230

[22] Jiang Y, Zhang NR, Li M. 2017 SCALE: modeling allele-specific gene expression by single-cell RNA sequencing. Genome Biol. **18**, 74. (10.1186/s13059-017-1200-8) 28446220 PMC5407026

[23] Zhang J, Zhou T. 2014 Promoter-mediated transcriptional dynamics. Biophys. J. **106**, 479-488. (10.1016/j.bpj.2013.12.011) 24461023 PMC3907263

[24] Suter DM, Molina N, Gatfield D, Schneider K, Schibler U, Naef F. 2011 Mammalian genes are transcribed with widely different bursting kinetics. Science **332**, 472-474. (10.1126/science.1198817) 21415320

[25] Van Kampen NG. 1992 Stochastic processes in physics and chemistry. Amsterdam, The Netherlands: Elsevier.

[26] Fuda NJ, Ardehali MB, Lis JT. 2009 Defining mechanisms that regulate RNA polymerase II transcription *in vivo*. Nature **461**, 186-192. (10.1038/nature08449) 19741698 PMC2833331

[27] Harper CV et al. 2011 Dynamic analysis of stochastic transcription cycles. PLoS Biol. **9**, e1000607. (10.1371/journal.pbio.1000607) 21532732 PMC3075210

[28] Zhang J, Zhou T. 2019 Markovian approaches to modeling intracellular reaction processes with molecular memory. Proc. Natl Acad. Sci. USA **116**, 23 542-23 550. (10.1073/pnas.1913926116) 31685609 PMC6876203

[29] Stumpf PS et al. 2017 Stem cell differentiation as a non-Markov stochastic process. Cell Syst. **5**, 268-282. (10.1016/j.cels.2017.08.009) 28957659 PMC5624514

[30] Voss TC, Hager GL. 2014 Dynamic regulation of transcriptional states by chromatin and transcription factors. Nat. Rev. Genet. **15**, 69-81. (10.1038/nrg3623) 24342920 PMC6322398

[31] Pedraza JM, Paulsson J. 2008 Effects of molecular memory and bursting on fluctuations in gene expression. Science **319**, 339-343. (10.1126/science.1144331) 18202292

[32] Jia T, Kulkarni RV. 2011 Intrinsic noise in stochastic models of gene expression with molecular memory and bursting. Phys. Rev. Lett. **106**, 058102. (10.1103/PhysRevLett.106.058102) 21405439

[33] Zhang J, Chen L, Zhou T. 2012 Analytical distribution and tunability of noise in a model of promoter progress. Biophys. J. **102**, 1247-1257. (10.1016/j.bpj.2012.02.001) 22455907 PMC3309289

[34] Zhou T, Zhang J. 2012 Analytical results for a multistate gene model. SIAM J. Appl. Math. **72**, 789-818. (10.1137/110852887)

[35] Zoller B, Nicolas D, Molina N, Naef F. 2015 Structure of silent transcription intervals and noise characteristics of mammalian genes. Mol. Syst. Biol. **11**, 823. (10.15252/msb.20156257) 26215071 PMC4547851

[36] Desai RV et al. 2021 A DNA repair pathway can regulate transcriptional noise to promote cell fate transitions. Science **373**, eabc6506. (10.1126/science.abc6506) 34301855 PMC8667278

[37] Rodriguez J, Ren G, Day CR, Zhao K, Chow CC, Larson DR. 2019 Intrinsic dynamics of a human gene reveal the basis of expression heterogeneity. Cell **176**, 213-226. (10.1016/j.cell.2018.11.026) 30554876 PMC6331006

[38] Klindziuk A, Kolomeisky AB. 2018 Theoretical investigation of transcriptional bursting: a multistate approach. J. Phys. Chem. B **122**, 11 969-11 977. (10.1021/acs.jpcb.8b09676) 30481024

[39] Sepúlveda LA, Xu H, Zhang J, Wang M, Golding I. 2016 Measurement of gene regulation in individual cells reveals rapid switching between promoter states. Science **351**, 1218-1222. (10.1126/science.aad0635) 26965629 PMC4806797

[40] Fritzsch C, Baumgärtner S, Kuban M, Steinshorn D, Reid G, Legewie S. 2018 Estrogen-dependent control and cell-to-cell variability of transcriptional bursting. Mol. Syst. Biol. **14**, e7678. (10.15252/msb.20177678) 29476006 PMC5825209

[41] Daigle Jr BJ, Soltani M, Petzold LR, Singh A. 2015 Inferring single-cell gene expression mechanisms using stochastic simulation. Bioinformatics **31**, 1428-1435. (10.1093/bioinformatics/btv007) 25573914 PMC4492418

[42] Schwabe A, Rybakova KN, Bruggeman FJ. 2012 Transcription stochasticity of complex gene regulation models. Biophys. J. **103**, 1152-1161. (10.1016/j.bpj.2012.07.011) 22995487 PMC3446772

[43] Kumar N, Singh A, Kulkarni RV. 2015 Transcriptional bursting in gene expression: analytical results for general stochastic models. PLoS Comput. Biol. **11**, e1004292. (10.1371/journal.pcbi.1004292) 26474290 PMC4608583

[44] Zhang J, Zhou T. 2019 Stationary moments, distribution conjugation and phenotypic regions in stochastic gene transcription. Math. Biosci. Eng. **16**, 6134-6166. (10.3934/mbe.2019307) 31499756

[45] Stinchcombe AR, Peskin CS, Tranchina D. 2012 Population density approach for discrete mRNA distributions in generalized switching models for stochastic gene expression. Phys. Rev. E **85**, 061919. (10.1103/PhysRevE.85.061919) 23005139

[46] Shi C, Jiang Y, Zhou T. 2020 Queuing models of gene expression: analytical distributions and beyond. Biophys. J. **119**, 1606-1616. (10.1016/j.bpj.2020.09.001) 32966761 PMC7642270

[47] Shelansky R, Abrahamsson S, Doody M, Brown CR, Patel HP, Lenstra TL, Larson DR, Boeger H. 2022 A telltale sign of irreversibility in transcriptional regulation. *bioRxiv*. 10.1038/s41467-024-50419-5 PMC11266658 39043639

[48] Blake WJ, Balázsi G, Kohanski MA, Isaacs FJ, Murphy KF, Kuang Y, Cantor CR, Walt DR, Collins JJ. 2006 Phenotypic consequences of promoter-mediated transcriptional noise. Mol. Cell **24**, 853-865. (10.1016/j.molcel.2006.11.003) 17189188

[49] Dunham LS et al. 2017 Asymmetry between activation and deactivation during a transcriptional pulse. Cell Syst. **5**, 646-653. (10.1016/j.cels.2017.10.013) 29153839 PMC5747351

[50] Lagha M et al. 2013 Paused Pol II coordinates tissue morphogenesis in the *Drosophila* embryo. Cell **153**, 976-987. (10.1016/j.cell.2013.04.045) 23706736 PMC4257494

[51] Coulon A, Ferguson ML, de Turris V, Palangat M, Chow CC, Larson DR. 2014 Kinetic competition during the transcription cycle results in stochastic RNA processing. Elife **3**, e03939. (10.7554/eLife.03939) 25271374 PMC4210818

[52] Coleman RA et al. 2017 p53 dynamically directs TFIID assembly on target gene promoters. Mol. Cell. Biol. **37**, e00085-17. (10.1128/MCB.00085-17) 28416636 PMC5472829

[53] Floyd DL, Harrison SC, Van Oijen AM. 2010 Analysis of kinetic intermediates in single-particle dwell-time distributions. Biophys. J. **99**, 360-366. (10.1016/j.bpj.2010.04.049) 20643053 PMC2905077

[54] Braichenko S, Holehouse J, Grima R. 2021 Distinguishing between models of mammalian gene expression: telegraph-like models versus mechanistic models. J. R. Soc. Interface **18**, 20210510. (10.1098/rsif.2021.0510) 34610262 PMC8492181

[55] Cox DR. 1955 The analysis of non-Markovian stochastic processes by the inclusion of supplementary variables. Cambridge, UK: Cambridge University Press.

[56] Alfa AS, Rao TS. 2000 Supplementary variable technique in stochastic models. Probab. Eng. Inf. Sci. **14**, 203-218. (10.1017/S0269964800142068)

[57] Zhang J, Nie Q, Zhou T. 2016 A moment-convergence method for stochastic analysis of biochemical reaction networks. J. Chem. Phys. **144**, 194109. (10.1063/1.4950767) 27208938 PMC4874931

[58] Franks JJ. 2020 Handbook of approximate Bayesian computation. Boca Raton, FL: CRC Press.

[59] Sisson SA, Fan Y, Tanaka MM. 2007 Sequential Monte Carlo without likelihoods. Proc. Natl Acad. Sci. USA **104**, 1760-1765. (10.1073/pnas.0607208104) 17264216 PMC1794282

[60] Zechner C, Ruess J, Krenn P, Pelet S, Peter M, Lygeros J, Koeppl H. 2012 Moment-based inference predicts bimodality in transient gene expression. Proc. Natl Acad. Sci. USA **109**, 8340-8345. (10.1073/pnas.1200161109) 22566653 PMC3361437

[61] Fröhlich F, Thomas P, Kazeroonian A, Theis FJ, Grima R, Hasenauer J. 2016 Inference for stochastic chemical kinetics using moment equations and system size expansion. PLoS Comput. Biol. **12**, e1005030. (10.1371/journal.pcbi.1005030) 27447730 PMC4957800

[62] Ellison AM. 1987 Effect of seed dimorphism on the density-dependent dynamics of experimental populations of *Atriplex triangularis* (Chenopodiaceae). Am. J. Bot. **74**, 1280-1288. (10.1002/j.1537-2197.1987.tb08741.x)

[63] Lenive O, W Kirk PD, H Stumpf MP. 2016 Inferring extrinsic noise from single-cell gene expression data using approximate Bayesian computation. BMC Syst. Biol. **10**, 81. (10.1186/s12918-016-0324-x) 27549182 PMC4994381

[64] Ochab-Marcinek A, Tabaka M. 2010 Bimodal gene expression in noncooperative regulatory systems. Proc. Natl Acad. Sci. USA **107**, 22 096-22 101. (10.1073/pnas.1008965107) 21135209 PMC3009792

[65] Sarkar A, Stephens M. 2021 Separating measurement and expression models clarifies confusion in single-cell RNA sequencing analysis. Nat. Genet. **53**, 770-777. (10.1038/s41588-021-00873-4) 34031584 PMC8370014

[66] Larsson AJ, Ziegenhain C, Hagemann-Jensen M, Reinius B, Jacob T, Dalessandri T, Hendriks G-J, Kasper M, Sandberg R. 2021 Transcriptional bursts explain autosomal random monoallelic expression and affect allelic imbalance. PLoS Comput. Biol. **17**, e1008772. (10.1371/journal.pcbi.1008772) 33690599 PMC7978379

[67] Larsson AJ, Coucoravas C, Sandberg R, Reinius B. 2019 X-chromosome upregulation is driven by increased burst frequency. Nat. Struct. Mol. Biol. **26**, 963-969. (10.1038/s41594-019-0306-y) 31582851

[68] Tantale K et al. 2021 Stochastic pausing at latent HIV-1 promoters generates transcriptional bursting. Nat. Commun. **12**, 4503. (10.1038/s41467-021-24462-5) 34301927 PMC8302722

[69] Engl C, Jovanovic G, Brackston RD, Kotta-Loizou I, Buck M. 2020 The route to transcription initiation determines the mode of transcriptional bursting in *E. coli*. Nat. Commun. **11**, 2422. (10.1038/s41467-020-16367-6) 32415118 PMC7229158

[70] Dobrzyński M, Bruggeman FJ. 2009 Elongation dynamics shape bursty transcription and translation. Proc. Natl Acad. Sci. USA **106**, 2583-2588. (10.1073/pnas.0803507106) 19196995 PMC2650307

[71] Keene J et al. 2001 As examples accumulate of both ARE-bearing stable mRNAs and labile mRNAs lacking AREs, the ARE dogma has incrementally given way to alternative bona. Nat. Rev. Mol. Cell Biol. **2**, 237-246. (10.1016/j.molcel.2008.01.007) 11283721

[72] Decker CJ, Parker R. 1993 A turnover pathway for both stable and unstable mRNAs in yeast: evidence for a requirement for deadenylation. Gene Dev. **7**, 1632-1643. (10.1101/gad.7.8.1632) 8393418

[73] Gorin G, Pachter L. 2022 Modeling bursty transcription and splicing with the chemical master equation. Biophys. J. **121**, 1056-1069. (10.1016/j.bpj.2022.02.004) 35143775 PMC8943761

[74] Fu X, Patel HP, Coppola S, Xu L, Cao Z, Lenstra TL, Grima R. 2022 Quantifying how post-transcriptional noise and gene copy number variation bias transcriptional parameter inference from mRNA distributions. Elife **11**, e82493. (10.7554/eLife.82493) 36250630 PMC9648968

[75] Kim JK, Kolodziejczyk AA, Illicic T, Teichmann SA, Marioni JC. 2015 Characterizing noise structure in single-cell RNA-seq distinguishes genuine from technical stochastic allelic expression. Nat. Commun. **6**, 8687. (10.1038/ncomms9687) 26489834 PMC4627577

[76] Gorin G, Fang M, Chari T, Pachter L. 2022 RNA velocity unraveled. PLoS Comput. Biol. **18**, e1010492. (10.1371/journal.pcbi.1010492) 36094956 PMC9499228

[77] Luo S, Zhang Z, Wang Z, Yang X, Chen X, Zhou T, Zhang J. 2023 Inferring transcriptional bursting kinetics from single-cell snapshot data using a generalized telegraph model. Figshare. (10.6084/m9.figshare.c.6486197) PMC10073913 37035293

